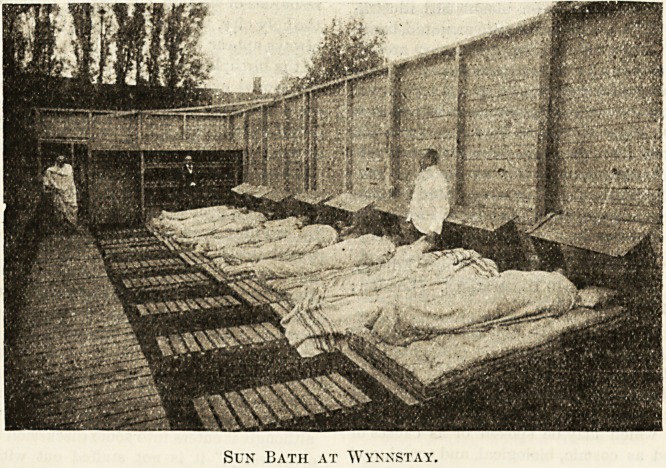# Wynnstay Sanatorium, Burgess Hill, Sussex

**Published:** 1901-12-07

**Authors:** 


### The Institutional Workshop.

### WYNNSTAY SANATORIUM, BURGESS HILL, SUSSEX.

This is a fine modern mansion within a short walk of Burgess Hill Station, on the London, Brighton and South Coast Railway. It was intended as a private residence, and hence it has nothing of the institutional appearance about it. Everything connected with it has been carried out in good taste, and the alterations rendered necessary to make it suitable for a sanatorium have not interfered with the harmony of the original design.

The baths and the electric installations for the various apparatus now used in the treatment of many diseases, have been fitted up at great cost and are in every direction of the most satisfactory and modern description. In the old system of electric bath the patient was wholly immersed in the water, and it was impossible to know how much of the current was actually received by the patient. In the system employed at Wynnstay, and known as that of Schnée, the patient sits in a chair with his legs and arms in separate baths, and the current can therefore be passed to or from any limb, and it can be accurately adjusted and gauged.

This four-celled system, as it is called, has certainly great advantages over the old systems in which it was found impossible to make strong currents of electricity pass through the body; and, moreover, by the four-celled system, such substances as iodine, lithium, etc., can be dissolved in the water, and may, perhaps, to some extent supplement the action of the electric current.

Here, too, we find Haelberg's electric vibrator, an instrument capable of giving 6,000 vibrations a minute, and used in such affections as lumbago, articular rheumatism, dilated stomach, etc.

The radiant heat and light bath at Wynnstay is one in which the patient sits, and in which, excepting his head, he is surrounded with incandescent lamps. There are 40 of these lamps, each of 16 candle-power. Part of the woodwork of this bath has been removed, and the opening glazed. Through this, the light, coloured or otherwise, from a powerful arc radiator can be turned on to the patient when he is in the bath, and thus a double benefit may, it is hoped, result from this combination. This system is used in chronic arthritic affections among others. Another special apparatus is that of Otter. This instrument is a pneumatic vibrator, and is employed in deafness resulting from disease of the middle ear. In certain cases it would seem that improvement follows its use.

The baths are of every, or almost every, description. There are Russian baths, sitting steam baths, reclining baths, douches, medicated steam inhalations, and many other varieties. It is worthy of a note that the men's baths are separate from the ladies'.

In the grounds there is a sun bath. This consists of a shed open to the sky, but surrounded by high wooden walls. It is about 35 feet long, and is provided with a series of folding bedsteads on which the patients lie extended, and in which only the head is protected from the direct action of the sun's rays. Part of this shed is used as an air bath. Dressing-rooms and shower baths are attached to the shed.

Plans for the erection of eight open-air châlets have been made, and these are to be proceeded with immediately They are intended for the treatment of anæmia, neurasthenia and insomnia. They are in design somewhat similar to the kiosks in use at consumption sanatoria; but phthisical cases would not be admitted at Wynnstay.

Sun Bath at Wynnstay.

In the hall we noticed a Rossbach's breathing chair. This is a contrivance for the treatment of emphysema and asthma, and it has features which incline us to believe that it might be useful in some instances of these diseases. There are also slings for carrying out the "suspension" treatment of locomotor ataxia.

It will be gathered from what we have said that this sanatorium is unusually well equipped in every way. In addition to what may be designated the more purely medical aspect of the treatment of disease, it is evident that the general aim of the institution is to combine home comforts with a carefully regulated system of everyday life which tends to invigorate the whole system of the patient, promotes recovery from disease, and enables him to resist it subsequently. With these objects Swedish exercises are employed three times a day for 10 minutes at a time. Early hours are insisted on, and alcohol forms no part of the diet.

The Sanatorium is intended to be as much as possible for *bonâ fide* patients; and, although it would be impossible to refuse admission to people who are well, such guests, if any, must conform to the régime laid down for patients.

The principal diseases with the treatment of which this Sanatorium is identified are neurasthenia, neuralgia, sciatica, insomnia, chronic bronchitis, emphysema, dilated stomach, gout, rheumatism, heart disease, and many other diseases of chronic type. The institution is entirely under the control of Dr. Whitby.

## Figures and Tables

**Figure f1:**